# Feasibility and acceptability of commonly used screening instruments to identify frailty among community-dwelling older people: a mixed methods study

**DOI:** 10.1186/s12877-020-01551-6

**Published:** 2020-04-22

**Authors:** Rachel C. Ambagtsheer, Mandy M. Archibald, Michael Lawless, Alison Kitson, Justin Beilby

**Affiliations:** 1 National Health and Medical Research Council Centre of Research Excellence in Trans-Disciplinary Frailty Research to Achieve Healthy Ageing, Adelaide, Australia; 2 Torrens University Australia, GPO Box 2025, Adelaide, SA 5000 Australia; 3 College of Nursing and Health Sciences, Flinders University, Adelaide, Australia; 4 College of Nursing, Rady Faculty of Health Sciences, University of Manitoba, Winnipeg, Manitoba Canada

**Keywords:** (MESH): frailty, Aged, 80 and over, Geriatric assessment, Primary health care, Mass screening

## Abstract

**Background:**

Frailty exposes older people to an elevated risk of a range of negative outcomes. Emerging evidence that frailty can be effectively treated within community settings has stimulated calls for more proactive screening within primary care. Assessing feasibility is a critical preliminary step in assessing the efficacy of interventions such as screening. However, few studies have explored the feasibility and acceptability of administering frailty screening instruments within general practice, and even fewer have incorporated patient perspectives. Our study had three objectives: To 1) assess overall feasibility of the instruments (completion time and rate); 2) assess patient acceptability towards the instruments; and 3) assess the feasibility and acceptability of the instruments to administering nurses.

**Methods:**

The feasibility and acceptability of several frailty screening instruments (PRISMA-7, Edmonton Frail Scale, FRAIL Scale Questionnaire, Gait Speed, Groningen Frailty Indicator, Reported Edmonton Frail Scale and Kihon Checklist) was explored within the context of a larger diagnostic test accuracy (DTA) study. Completion time and rate was collected for all participants (*N* = 243). A sub-sample of patients (*n* = 30) rated each instrument for ease of completion and provided comment on perceived acceptability. Lastly, five of six administering nurses involved in the DTA study participated in semi-structured face-to-face interviews, rating the instruments against several feasibility and acceptability criteria (time, space, equipment, skill required to implement, acceptability to patients and nurses, ease of scoring) and providing comment on their responses.

**Results:**

The PRISMA-7 returned the highest overall feasibility and acceptability, requiring minimal space, equipment, skills and time to implement, and returning the fastest completion rate and highest patient and nurse acceptability rating. All screening instruments were faster to implement than the two reference standards (Fried’s Frailty Phenotype and Frailty Index). Self-administered instruments were subject to lower rates of completion than nurse-administered instruments.

**Conclusions:**

This study has demonstrated that a number of commonly used frailty screening instruments are potentially feasible for implementation within general practice. Ultimately, more research is needed to determine how contextual factors, such as differences in individual patient and clinician preferences, setting and system factors, impact on the feasibility of screening in practice.

## Background

Frailty is a key challenge facing health systems across the globe as world population ageing progresses [1–3]. Defined as a geriatric condition characterised by increased susceptibility to external stressors [3–5], frailty brings an elevated risk of negative outcomes for older people, including falls, hospitalisation, residential care admission and mortality [6–9]. However, emerging evidence that frailty is a dynamic state able to be altered with appropriate intervention, rather than an incontrovertible consequence of old age, has led to increased calls for early detection and treatment [4, 10]. Primary care settings, and general practice in particular, are frequently asserted as ideal sites for identification and management of frailty [11–13]. Yet frailty awareness within primary care remains low in many countries [14], and a number of barriers are evident.

Among these barriers is the fact that, despite the development of numerous frailty screening instruments [3, 15, 16], commensurate efforts towards determining the feasibility of instrument implementation is lacking [17–19]. Feasibility studies address the question of whether an intervention can work in practice, and typically focus on process considerations rather than outcomes [20]. Only a small number of studies have addressed the feasibility of frailty screening within general practice settings [21–26], and these have generally addressed electronic means of frailty identification and assessment rather than administered instruments. Those which have assessed administered instruments have tended to focus on a single instrument [24, 25], rather than comparing multiple instruments simultaneously. Although one study did assess the feasibility of two administered instruments (PRISMA-7 and Gait Speed) alongside an electronic Frailty Index (eFI), it did not incorporate both patient and provider perspectives [26]. Consequently, the aim of our study was to assess the feasibility and acceptability of several widely used frailty screening instruments within a general practice context. Within this aim, we had several objectives:

1. To assess the feasibility of the instruments with regard to time to complete and completion rates;
2. To assess the acceptability of the instruments to patients; and.
3. To assess the feasibility and acceptability of the instruments to the administering practice nurses.

## Methods

### Design

A feasibility and acceptability evaluation was incorporated within a larger study determining the diagnostic test accuracy (DTA) of several commonly used frailty screening instruments [19] in three South Australian general practices between August 2017 and June 2018. The feasibility and acceptability components were comprised of three sub-studies; (a) a quantitative analysis of completion time and missing rate based on the DTA results; (b) a mixed-methods analysis of patient acceptability based on a sub-sample of the DTA study sample (*n* = 30) and (c) a mixed-methods evaluation of nurse feasibility and acceptability conducted via face-to-face interviews with administering nurses (*n* = 5). The Torrens University Higher Research Ethics Committee granted approval for the study (HREC 10/17). All participants gave written, informed consent. More detail regarding the study setting can be found in the published protocol [19].

### Sub-study 1: quantitative analysis of completion time and rate

#### Participants

The recruitment process for practices and participants within the DTA study has been described in detail elsewhere [19]. Briefly, primary care practices were purposively selected to meet study design requirements, and participants were recruited using a random integer generator from a full list of eligible patients generated by the practice staff [27]. Patients were eligible to participate if they were English speaking, community-dwelling patients of the practice and aged 75 years or over as of 30 June 2017. Patients receiving palliative care were ineligible to participate.

#### Data collection and analysis

Data collection occurred during regular face-to-face appointments at the practice. Patients completed five nurse-administered frailty screening tests (Edmonton Frail Scale (EFS) [28], FRAIL Scale Questionnaire (FQ) [29], Gait Speed (GST) [11, 30, 31], Groningen Frailty Indicator (GFI) [32] and PRISMA-7 [33]) and two self-administered tests incorporated within a written survey (the Reported Edmonton Frail Scale (REFS) [34] and the Kihon Checklist (KC) [35]). Screening instruments were administered in a random order. Instruments were selected on the basis of 1) appropriateness for the Australian context (thereby excluding instruments such as the Tilburg Frailty Indicator, which references European concepts) 2) minimal clinician judgement, in recognition of the fact that it was likely not general practitioners who would ultimately be administering the instruments and 3) frequency of use within the reported frailty literature. In addition, a member of the research team (RA) administered two reference standards: 1) Fried’s Frailty Phenotype (FP) [6], a measure including gait speed, grip strength, self-reported exhaustion, weight loss and physical activity [6] and 2) a modified (39 item) version of the Adelaide Frailty Index (FI), a self-reported Frailty Index [36] covering multiple dimensions. The frailty thresholds for the reference standards were as follows: for FP, 1–2 criteria = pre-frail, 3 or more = frail [6]; for FI, pre-frail was defined as > 0.1 to ≤0.21 and frail (> 0.21) [37]. All thresholds for instruments and reference standards, along with key feasibility characteristics, are shown in Table 1.

**Table 1:** Frailty Screening Instruments and Reference Standards Included in the Study

Instrument	Frailty Threshold	Training required to administer	Training required to score	Equipment required	Physical space required
**Index Tests (Nurse-Administered)**					
Edmonton Frail Scale	≥ 8 points	Use of stopwatch (TUG); Distance set up	Clock test; TUG; reverse scoring	Stopwatch; Distance Measure; Tape	3 m straight corridor
FRAIL Questionnaire	≥ 3 points	None	Minimal	None	Minimal
Gait Speed	≤ 0.8 m/s	Use of stopwatch; Distance set up	Metres/second calculation	Stopwatch; Distance Measure; Tape	4 m straight corridor
Groningen Frailty Indicator	≥ 4 points	None	Reverse scoring	None	Minimal
PRISMA-7	≥ 3 points	None	Minimal	None	Minimal
**Index Tests (Self-Administered)**					
Kihon Checklist	≥ 7 points	None	BMI Calculation	None	Minimal
Reported Edmonton Frail Scale	≥ 8 points	None	Clock test	None	Minimal
**Reference Standards**					
Frailty Index (Self-Reported)	> = 0.21	None	Minimal	None	Minimal
Frailty Phenotype	3 of 5 criteria	Use of stopwatch (Gait Speed); Distance set up; Use of dynamometer (Grip Strength)	Extensive (PASE measure, Gait Speed incl. Height; Grip Strength incl. BMI)	Stopwatch; Dynamometer; Scales; Height measure	15 ft (4.6 m) straight corridor (Gait Speed); area to assess height, weight, conduct grip strength assessment

Abbreviations: *BMI* Body Mass Index, *PASE* Physical Activity Scale for the Elderly, *TUG* Timed Up and Go

After administration, the practice nurse or researcher noted the time in minutes to administer the instrument on a 5-point scale (Less than 5; 5 to < 10; 10 to < 20; 20 to < 40; 40 min or greater). Where participants did not complete the instrument, the reason for non-completion was recorded. In regard to the written survey, nurses were instructed to leave the two self-administered frailty instruments unchecked for completion in order to determine completion rates. Given the inclusion of these items within a larger survey, it was not possible to collect completion times for REFS and KC.

All quantitative data were initially entered into Microsoft Excel and then analysed using descriptive statistics in IBM SPSS v.25.

### Sub-study 2: patient acceptability component

#### Participants

At the start of the appointment, practice nurses invited patients to answer additional questions regarding their perceived acceptability of the screening tools. We aimed to recruit a minimum of a 10% (*n* = 25) sample, based on established practice within the literature [38] and which reflects the minimum recommended number of participants for a feasibility study addressing instrumentation [39].

#### Data collection and analysis

Patients who consented to complete the acceptability survey were asked to rate each instrument immediately after completion on a Likert scale from 1 (‘Very Easy’) to 5 (‘Very Hard’) in response to the question, ‘How easy was this task to complete?’ Practice nurses documented patients’ verbal comments regarding instrument acceptability. The acceptability of the two self-administered instruments was incorporated within the written survey and followed the same format, although in this instance, patients wrote rather than spoke their responses.

All quantitative and qualitative results were entered and analysed in Microsoft Excel. Quantitative results were analysed using descriptive statistics. A content analysis based on these responses was conducted by RA to determine themes identified by participants and then were shared with another research team member (ML) to verify the results [40].

### Sub-study 3: administering nurse feasibility and acceptability component

#### Participants

Practice nurses who administered the screening instruments were asked to participate in an individual interview focusing on their impressions of the screening tools with a member of the research team (RA), with the exception of the nurse from the withdrawn site. The overall number of participating nurses was relatively low (*n* = 5); however, given the pivotal role of clinical staff in the ultimate success of interventions [41], it was considered imperative that their perspectives be canvassed and presented here, even if only for illustrative purposes. There are a range of reasons why providing this information might prove useful to future researchers and clinicians [38], not least of which is to inform the future implementation of these tools in clinical practice.

#### Data collection and analysis

Participating nurses were interviewed by a member of the research team (RA) within two months of DTA data collection. Nurses rated each instrument on a scale of 1 (low) to 10 (high) against several feasibility and acceptability criteria. The criteria included time to implement, space required, skills required, equipment required, acceptability to patient, and acceptability to administering nurse, and were based on the feasibility.

areas identified by Orsmond and Cohen that were most pertinent to the research objectives [20], together with alignment with the most common feasibility elements explored in previous frailty research [24–26]. Participants were also asked to verbally explain reasons for their ratings.

Interviews were audio and/or video recorded and professionally transcribed verbatim. Transcripts were analysed independently by two researchers (RA and ML) within Microsoft Excel using a qualitative descriptive approach informed by thematic analysis. Transcripts were read repeatedly to generate initial codes using a combined deductive/inductive approach. This included a priori codes reflecting the six feasibility dimensions outlined above and sub-codes within each criterion. Sub-codes were inductively derived and generally reflected participants’ explanation of their feasibility ratings RA and ML then compared and refined the codes to obtain an agreed upon coding framework, which was applied to the transcripts. Codes were then analysed to develop a set of representative themes. The final interpretation of results and selection of indicative quotations from participants was decided through consultation with the wider research team.

## Results

### Sub-study 1: participant recruitment and characteristics

We recruited 243 participants into the screening study (Table 2). Participants had a median age of 79 years (IQR 6.0); just over half were female (55.6%, *n* = 135). Approximately one-third (31.5%, *n* = 76) lived alone; 42.9% had a high school education or higher (*n* = 103). Very few (1.7%, *n* = 4) spoke a language other than English at home. Frailty prevalence was 17.2% (*n* = 41) according to FP and 50.0% (*n* = 120) according to FI.

**Table 2:** Participant characteristics

	Participants (*N* = 243)
Age at appointment (y): Median (IQR)	79.0 (6.0)
Gender	
Male	108 (44.4)
Female	135 (55.6)
Lives alone	76 (31.5)
High school education or higher	103 (42.9)
Speaks language other than English at home	4 (1.7)
Attends rural practice	120 (49.4)
Takes 5+ medications	97 (39.9)
Frailty status (FP)	
Robust	61 (25.6)
Pre-frail	136 (57.1)
Frail	41 (17.2)
Frailty status (FI)	
Not frail	51 (21.3)
Pre-frail	69 (28.7)
Frail	120 (50.0)

Note. Abbreviations: *FI* Frailty Index, *FP* Frailty Phenotype, *IQR* Inter-Quartile Range. Missing data excluded from table: Age (*n* = 2); Living arrangements (*n* = 2), Education (*n* = 3), Language spoken (*n* = 1), Polypharmacy (*n* = 2), Frailty status (FP) (*n* = 5), Frailty status (FI) (*n* = 3)

**Fig. 1:**
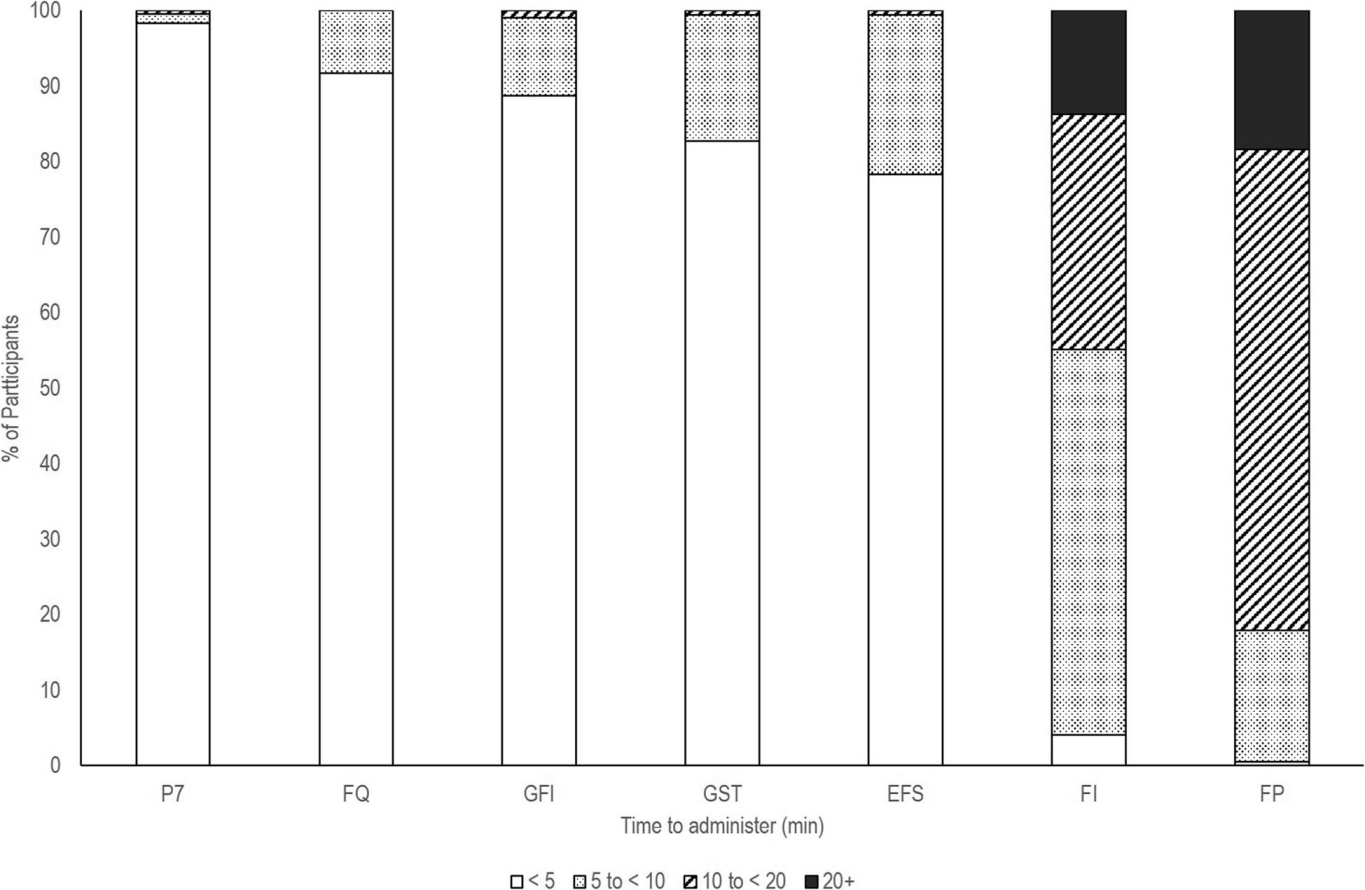
Time to administer instruments (minutes). Note: P7: PRISMA-7, FQ: Frail Questionnaire, GFI: Groningen Frailty Indicator, GST: Gait Speed Test, EFS: Edmonton Frail Scale, FI: Frailty Index, FP: Frailty Phenotype

All frailty screening instruments analysed were implemented faster on average than the two reference standards (Fig. 1). For virtually all participants, each instrument was administered in under 10 min, whereas in the case of the reference standards, this was true for only 18.0% (FP) and 55.2% (FI) of participants. The PRISMA-7 and FRAIL questionnaire were the fastest instruments, with both administered to over 90.0% of participants in under five minutes.

#### Completion rate

On the whole, nurse or researcher-administered instruments performed better than self-administered instruments with respect to rate of completion (Table 3, see also Supplement 1). Complete data was obtained for over 96% of participants for all administered instruments, whereas for the two self-administered instruments, completion rate was 68.3 and 90.9% for KC and REFS respectively. The most frequent non-completed items for KC were self-reported height (17.3%) and self-reported weight (9.5%); these items were.

**Table 3:** Item Completion for index tests and reference standards (*N* = 243)

Instrument	No. total individual items required for calculation of instrument^a^	Participants with all data items complete n (%)	Participants with > 10% data items missing n (%)
Index tests: Nurse-Administered			
Edmonton Frail Scale	11	238 (97.9)	1 (0.4)
Frail Questionnaire	16	237 (97.5)	2 (0.8)
Gait Speed	2	239 (98.4)	4 (1.6)
Groningen Frailty Index	15	243 (100.0)	0 (0.0)
PRISMA-7	7	240 (98.8)	3 (1.2)
Index tests: Self-Administered			
Kihon Checklist	26	166 (68.3)	14 (5.8)
Reported Edmonton Frail Scale	13	221 (90.9)	6 (2.5)
Reference standards			
Modified Adelaide Frailty Index	39	240 (98.8)	1 (0.4)
Frailty Phenotype	15	235 (96.7)	4 (1.7)

Note. ^a^ Frail Questionnaire: Includes weight one year ago, weight today as 2 items. Kihon Checklist: Includes self-reported height and weight for BMI calculation as 2 items. Gait Speed: Measured over two attempts = 2 items. Frailty Phenotype: Shrinking (1 item); Exhaustion (2 items); Gait Speed (1 item); Grip Strength: 3 attempts (3 items) plus height, weight, gender (3 items), Physical Activity (5 items)

the most significant contributing factors in the comparatively low rate of self-completion for KC.

### Sub-study 2: acceptability to patients

We recruited 30 patient participants from the screening sample to complete the acceptability questionnaire. Each rated 7 screening instruments, resulting in 210 unique acceptability-related data points. In general, all screening instruments were rated as easy to complete by patients, with 93.5% of responses rating instruments as Very Easy/Easy to complete, and the median and mode for all instruments also rated as either Very Easy/Easy. However, some variation was observed, as shown in Table 4. Overall, the P7 was found to be most acceptable to patients, with a median and mode rating of 1 (Very Easy). All patients rated it as Easy/Very Easy on the Likert scale. Gait speed received the lowest overall rating, with 86.2% of patients rating it as Very Easy/Easy.

**Table 4:** Joint display of patient acceptability ratings (*n* = 30)

Instrument	Very Easy/ Easy n (%)	Participant comments (Easy/Very Easy)	Neutral/ Difficult n (%)	Participant comments (Neutral/Difficult)
EFS	28 (96.6)	•Not as quick as I used to be •It did show me what I can’t do anymore •Easy but you need to think first. •Easy enough	1 (3.4)	•Found it frustrating. The clock face. I don’t like these sorts of puzzles!
FQ	27 (96.4)	•I had no difficulty. I found it interesting. •A bit of thinking •Nothing hard about the questions	1 (3.6)	•n.a.
GAIT	25 (86.2)	•Easy to complete •No issues to do •Simple task	4 (13.8)	•It’s not easy, had to slow down, painful hip •After sitting for a while, my legs cramp. I would have been quicker if I had not been sitting.
GFI	26 (89.7)	•Some questions could go either way. Sometimes yes/ sometimes no. •Once I worked out the questions •Went quickly enough •No problems completing this	3 (10.3)	•You have to think - it can never be cut and dried. •OK question - have to stop and think about questions as don’t normally think about these situations
KC	25 (89.3)	•I thought it was challenging, but rather appropriate and it made me realise how fortunate I am to be able to enjoy my activities, and the people in my life •I’m always thankful for anything I can do •Interesting - covered a whole gamut of day to day living	3 (10.7)	•Mostly easy, some didn’t apply •My life has been very difficult over the last eighteen months •Don’t normally go out much; socialise within street
P7	30 (100.0)	•No worries answering questions •Simple and easy	0 (0.0	•n.a.
REFS	25 (96.2)	•Hated it. Don’t like this - not many people would. •Had to think more about this one. Trying to do it a bit too quickly. Made me think. •Didn’t have any problems. A long list of what you can’t do. •Quite reasonable things to ask older persons •Clock was a bit confusing - wasn’t sure of what the question meant re the numbering around the edge	1 (3.8)	•n.a.
**ALL**	**186 (93.5)**		**13 (6.5)**	

Abbreviations: *P7* PRISMA-7, *FQ* Frail Questionnaire, GAIT: Gait Speed Test, *GFI* Groningen Frailty Indicator, *GST* Gait Speed Test, *EFS* Edmonton Frail Scale, *KC* Kihon Checklist, *REFS* Reported Edmonton Frail Scale

Thematic analysis of participant responses to the open-ended question (*How did you find this task?*) resulted in five acceptability-related categories including: Easy to Use (49.5% of responses), Reasonable (11.9%), Stimulated Self-Reflection (11.0%), Challenging (7.1%) and Inapplicable (1.4% - indicating that the instrument included ambiguous questions or ones that did not apply to them). Approximately one-quarter (24.8%) of responses were blank. On the whole, participants gave responses such as *‘Straightforward’* or *‘Easy’* that aligned with their Likert scale rating for the instrument. However, in some cases where participants had rated an instrument as easy to complete, their verbal response reflected low acceptability, as shown by the following response on the REFS: *“Hated it. Don’t like this – not many people would*.” Conversely, there was also a case where the opposite was true, wherein a participant commenting that a task was “*… mostly easy, some didn’t apply*” subsequently rated it as neutral on the Likert scale.

A number of participants commented that instrument completion stimulated an element of self-reflection or self-awareness. This was often couched positively or negatively and in relation to participants’ psychological response to being questioned about some aspect of their current health and function. For some, completing an instrument prompted a positive realisation of their own capacity and abilities. As one participant commented on the KC remarked “*I thought it was challenging, but rather appropriate and it made me realise how fortunate I am to be able to enjoy my activities, and the people in my life.”* However, for others, instrument completion led to a greater sense of awareness of their own limitations, as two participants noted, both in relation to the EFS: “*It did show me what I can’t do anymore”* and “*Not as quick as I used to be*.”

Of the participants reporting that instrument completion was challenging, most indicated the task was mentally challenging in the sense of having to think before responding to the question, as in the case of a participant commenting on the REFS: “*Had to think about one or two questions - i.e. can walk a distance but may need to stop and rest*.” Instruments perceived as challenging in this way included the REFS, FQ, KC and GFI. Others perceived instrument completion as physically challenging, with one participant noting during their experience of the GST, “*It’s not easy, had to slow down, painful hip*.” Instruments perceived as physically challenging were the GST and EFS, both of which incorporate a physical component.

### Sub-study 3: perceived feasibility and acceptability to administering nurses

Administering nurses (*n* = 5) participating in the feasibility component were all female, aged 45 years or over, and had over 20 years’ professional nursing experience. Overall, the PRISMA-7 was ranked most feasible and acceptable by nurses, scoring a median value of 10/10 for five of the six criteria (Table 5). Instruments identified by nurses as more time consuming included the GFI, KC, EFS, GST and REFS. Reasons expressed for rating instruments lower on time included patients needing time to think about questions, patients becoming confused by the task, needing time to explain instructions and addressing patient’s answers to health/psychosocial questions.

**Table 5:** Administering Nurse: Perceived Feasibility and Acceptability (Median Rating/10) by Instrument (*n* = 5)

Instrument	Time	Space	Equipment	Skill	Acceptability (Patient)	Acceptability (Nurse)
	Median (Q_1_ – Q_3_)	Median (Q_1_ – Q_3_)	Median (Q_1_ – Q_3_)	Median (Q_1_ – Q_3_)	Median (Q_1_ – Q_3_)	Median (Q_1_ – Q_3_)
P7	10.0 (9.5–10.0)	10.0 (8.25–10.0)	10.0 (9.5–10.0)	10.0 (9.5–10.0)	9.0 (7.5–10.0)	10.0 (8.5–10.0)
EFS	8.0 (6.5–8.0)	6.0 (5.5–7.0)	7.0 (5.5–8.0)	8.0 (6.5–10.0)	8.0 (5.5–8.0)	9.0 (8.5–9.5)
FQ	10.0 (8.5–10.0)	9.0 (8.25–10.0)	10.0 (9.5–10.0)	10.0 (9.5–10.0)	6.0 (6.0–9.5)	9.0 (8.5–10.0)
GST	8.0 (8.0–9.0)	7.0 (5.5–7.75)	8.0 (6.5–8.5)	9.0 (8–10.0)	7.0 (7.0–9.0)	10.0 (9.0–10.0)
GFI	8.0 (7.0–8.0)	9.0 (9.0–10.0)	10.0 (9.5–10.0)	10.0 (8.5–10.0)	8.0 (7.5–9.0)	9.0 (7.0–10.0)
KC	6.0 (5.5–7.5)	9.5 (9.0–10.0)	10.0 (9.5–10.0)	9.5 (7.5–10.0)	7.0 (6.5–7.5)	9.0 (7.5–10.0)
REFS	7.0 (6.0–7.5)	10.0 (9.0–10.0)	10.0 (9.5–10.0)	10.0 (9.25–10.0)^a^	8.0 (7.5–8.0)^a^	9.5 (8.25–10.0)

Abbreviations: *P7* PRISMA-7, *FQ* Frail Questionnaire, *GAIT* Gait Speed Test, *GFI* Groningen Frailty Indicator, *GST* Gait Speed Test, *EFS* Edmonton Frail Scale, *KC* Kihon Checklist, *REFS* Reported Edmonton Frail Scale. ^a^ Due to missing data, *n* = 4 for these rankings; data was adjusted accordingly

In addition, the requirement for sufficient space for patients to mobilise in order to complete specific instruments such as the EFS and GST tended to be perceived as a disadvantage by the nurses. Similarly, instruments not requiring specific equipment were ranked higher by nurses, with timed instruments requiring equipment such as a stopwatch ranked lower (EFS, GST). Most instruments were perceived as requiring minimal skill to administer, with several being allocated a median rating of 10/10.

In general, nurses found most of the instruments highly acceptable to administer; the median acceptability rating for all instruments ranged between 9.0 and 10.0. The P7 ranked highest overall with regard to patient acceptability. Most of the key issues identified by nurses related to specific items or instruments. However, a common theme was problems identified with the structure, design or wording of questions, making interpretation difficult for patients.

## Discussion

This study assessed the feasibility and acceptability of several commonly used screening instruments to identify frailty among community-dwelling older people within a general practice setting. We found that of all the instruments, the P7 returned the highest overall feasibility and acceptability results. It required minimal space, equipment, skills and time to implement and had the fastest completion rate of all the instruments analysed. Moreover, it had the highest acceptability rating for both patients and administering nurses, and was rated first for five of six individual criteria ranked by nurses. These findings for the P7 are consistent with respect to completion rate, skills, equipment, time to complete, space requirements and provider acceptability as a previous study conducted in general practice [26]. Recent research has also indicated that it returns relatively high diagnostic and predictive accuracy in primary care settings [13, 42–44], although more replication studies are needed to ensure generalisability. As a rapid screening instrument intended to be followed by more comprehensive assessment, the P7 may indeed prove viable in general practice settings, although more feasibility and acceptability testing under real world conditions is required.

Our findings lend weight to the viewpoint that rapid frailty screening instruments of the type included within this study are more practical to implement than reference standards within busy general practice settings. It has frequently been asserted within the frailty literature that GPs and their teams need to be able to implement simple, rapid frailty screening due to time constraints [4, 12, 25, 26, 42]. Our results show that screening instruments took consistently less time to implement than the reference standards, which in the case of the FP also required physical space and equipment that may not be readily available within most general practice settings. However, it is important to acknowledge that the instruments tested are intended primarily for *screening* purposes, while the reference standards are intended for *diagnosis*. Ideally, a positive result should be followed up by a lengthier, more comprehensive assessment in which frailty status is ascertained. Further, it is important to acknowledge that feasibility is both subjective and contingent on local context. Clinicians may differ over their perception of the ease with which even rapid frailty screening can be integrated into existing work flows, as demonstrated by prior research [25]. Several contextual factors can mediate clinician perception of feasibility, including factors relating to the patient, the clinician and the setting. For example, in our prior research on general practitioner attitudes towards frailty and frailty screening, age of the GP was identified as a potential factor shaping perceptions [45]. Further research is required to determine how contextual factors impact on the feasibility of screening in practice.

Self-administered instruments show potential as one means of offering greater efficiency within general practice settings, although their diagnostic accuracy and feasibility have not been well determined to date [46]. Time to complete was not collected for the two self-administered instruments assessed within this study (KC and REFS). However, the relatively high non-completion rate of these instruments in comparison with the nurse-administered instruments suggests that some adaptation to the approach adopted in our study may be needed to support their implementation in practice. Such adaptation could include having nurses check over the questionnaire after patient self-completion, modifying the instrument to remove problematic items, or supplementing missing data with information (e.g. recorded height and weight) drawn from the patient record. Given that any modification may alter the accuracy of the instruments, any such steps should be taken advisedly.

Our results suggest that the act of completing a frailty screening instrument stimulated self-reflection on both the potential and limits of their own functional ability among several patient participants. For some, this took the form of gratitude for what they were yet able to do; for others, the act of reflection led to a sense of realisation about what they could do no longer. These results would seem to support, at least in part, the potential for frailty to act as an opportunity for self-awareness and reflection, as sometimes espoused in frailty-focused editorials within the literature [47]. However, the extent to which this realisation leads to positive outcomes for older people is not yet clear. Certainly, the possibility that a frailty diagnosis may lead to formation of a negative self-concept within older people, and the ensuing negative impacts on health and wellbeing, has been widely acknowledged in the frailty literature [48–51]. Further, the prospect that even the act of completing a screening instrument may cause a similar effect has not been so readily recognised. More research is needed to untangle the complexities inherent in these, and similar, questions.

Several barriers and enablers for implementing frailty screening into clinical practice are implied by our analysis. Frequently, nurses in our study questioned whether patients had understood the full intent of a question and/or the instructions that accompanied a screening task. While there are a number of attributable causes for patients not fully comprehending an instrument (including ambiguous wording and question structures), one possibility is that the presence of mild cognitive impairment (MCI) in the patient may have impacted the results. This prospect underlines the importance of interpreting screening results in light of the patient’s medical history, and may signal a need to schedule a more comprehensive follow-up assessment following screening. Another barrier is the propensity for some patients to intentionally present an overly positive front, fearing the ramifications of a frailty diagnosis. In these cases, inclusion of objective measures such as a timed walk may give a more accurate depiction of true frailty status. Lastly, a number of enablers to support screening were implied by our discussions with administering nurses. These include the provision of training to ensure nurses are confident and comfortable when implementing instruments that address psychosocial concerns, and of resources and tools to support scoring of instruments where necessary.

### Strengths and limitations

To our knowledge, this is the most comprehensive study to date to explore the feasibility and acceptability of several widely used frailty screening instruments within a general practice setting. Further, in presenting both health service provider and patient perspectives of acceptability we have been able to address a significant imbalance within the existing literature and provide additional contextual evidence for providers seeking to implement responsive, person-centred care. In applying a mixed-methods approach, we have enabled a broader understanding of the feasibility and acceptability of instruments, as illustrated by the differentiation between ease of completion and acceptability identified when triangulating patient quantitative and qualitative responses.

However, there are some limitations to the study. It was not within the scope of the study to assess the feasibility and acceptability of the two reference standards (FP, FI). However, the limited data we were able to collect (time to administer and completion rate) was advantageous with respect to illustrating the time advantages of screening instruments over reference standards. Further, a full complement of results across the four sub-studies was not possible for all instruments included within the study. For example, it was not possible to individually time certain instruments (KC, REFS) due to their inclusion within the self-completed survey. This decision was taken to reduce participant burden and the overall complexity of the study. However, it is possible that this aspect of the study design led to lower feasibility rankings for these instruments, wherein nurses may have conflated the perceived time taken to complete individual instruments with the time to complete the whole survey.

Our sample of administering nurses was intentionally small in order to reduce practice burden. Although capturing administering nurse perspectives in our study added valuable insight into the feasibility of instruments in practice, generalisability to other contexts is limited, with more replication studies needed. Further, the delay in interview time of up to two months post-study may have impacted on accurate recall, although nurses were provided with a quick refresher on each instrument prior to interview commencement. Another limitation was that all of the administering nurses who participated in providing feasibility and acceptability data were female and highly experienced, thus constituting a relatively homogenous group. Further research is needed with a more diverse range of health service provider groups to provide a broader perspective. Our patient cohort was highly proficient in English; our results are likely not generalisable to patient cohorts with a large percentage of persons born in culturally and linguistically diverse contexts. We did not include all frailty screening instruments currently in use, for reasons of practicality and suitability to the Australian general practice context. Lastly, patient acceptability responses were paraphrased by the nurse for administered instruments but self-completed (handwritten by patients) for self-completed instruments. We expect that this may have somewhat reduced the richness of the acceptability data obtained for the administered instruments.

## Conclusion

This study explored the feasibility and acceptability of a range of commonly implemented frailty screening instruments within general practice across a number of dimensions. Our results suggest that several feasible and acceptable options for frailty screening implementation exist, with the PRISMA-7 performing best overall. However, besides feasibility and acceptability considerations, selection of the most appropriate instrument for implementation within a given primary care context requires careful deliberation on the purpose, setting and context for screening along with diagnostic and predictive accuracy. Sufficient initial time investment in this decision may hold the key to better identifying and treating frailty within primary care in the long term.

## Supplementary information

**Additional file 1.** Time to complete, Nurse-Administered Instruments and Reference Standards

## Data Availability

The data that support the findings of this study are available on request from the corresponding author [RA]. The data are not publicly available due to privacy and consent considerations.

## Abbreviations

BMI: Body Mass Index
DTA: Diagnostic Test Accuracy
eFI: Electronic Frailty Index
EFS: Edmonton Frail Scale
FI: Frailty Index
FP: Fried’s Frailty Phenotype
FQ: FRAIL Scale Questionnaire
GFI: Groningen Frailty Indicator
GP: General Practitioner
GST: Gait Speed Test
HREC: Higher Research Ethics Committee
IQR: Inter-Quartile Range
KC: Kihon Checklist
P7: Alternative abbreviation of PRISMA-7
PASE: Physical Activity Scale for the Elderly
PRISMA-7: Program of Research on Integration of Services for the Maintenance of Autonomy
REFS: Reported Edmonton Frail Scale
TUG: Timed Up and Go
