# Supplementary material for: Feasibility and acceptability of commonly used screening instruments to identify frailty among community-dwelling older people: a mixed methods study
Source: BMC Geriatr. 2020 Apr 22;20:152. doi: 10.1186/s12877-020-01551-6 (PMC7178952; doi:10.1186/s12877-020-01551-6)
Supplement: Supplementary file 1 — Additional file 1. Time to complete, Nurse-Administered Instruments and Reference Standards [file 12877_2020_1551_MOESM1_ESM.docx]

**Supplement 1: Time to complete, Nurse-Administered Instruments and Reference Standards**

| **Instrument** | **Time to administer (min)#**  **n=243 (% of row total)** | | | | |  |
| --- | --- | --- | --- | --- | --- | --- |
| **Index Tests (Nurse-Administered)** | **< 5** | **5 to < 10** | **10 to < 20** | **20+** |  | |
|  |  |  |  |  |  | |
| Edmonton Frail Scale | 170 (78.3) | 46 (21.2) | 1 (0.5) | 0 (0.0) |  | |
| FRAIL Questionnaire | 211 (91.7) | 19 (8.3) | 0 (0.0) | 0 (0.0) |  | |
| Gait Speed | 164 (82.8) | 33 (16.7) | 1 (0.5) | 0 (0.0) |  | |
| Groningen Frailty Indicator | 207 (88.8) | 24 (10.3) | 2 (0.9) | 0 (0.0) |  | |
| PRISMA-7 | 225 (98.3) | 3 (1.3) | 1 (0.4) | 0 (0.0) |  | |
|  |  |  |  |  |  | |
| **Reference Standards** |  |  |  |  |  | |
|  |  |  |  |  |  | |
| Frailty Index (Self-Reported) | 9 (4.1) | 112 (51.1) | 68 (31.1) | 30 (13.7) |  | |
| Frailty Phenotype | 1 (0.5) | 36 (17.5) | 131 (63.6) | 38 (18.4) |  | |
|  |  |  |  |  |  | |

# Excludes cases where time was not recorded (i.e. missing).
